# Promoting targeted heat early warning systems for at-risk populations

**DOI:** 10.1038/s41558-025-02374-2

**Published:** 2025-07-03

**Authors:** Fergus K. O’Connor, Mehak Oberai, Zhiwei Xu, Sebastian Binnewies, Shannon Rutherford, Robert D. Meade, Steven Baker, Ella Jackman, Connor Forbes, Aaron J. E. Bach

**Affiliations:** 1 School of Allied Health, Sport and Social Work, Griffith University, Gold Coast, Queensland, Australia; 2 School of Medicine and Dentistry, Griffith University, Gold Coast, Queensland, Australia; 3 School of Information and Communication Technology, Griffith University, Gold Coast, Queensland, Australia; 4 Department of Epidemiology, T.H. Chan School of Public Health, Harvard University, Boston, MA, USA; 5 The Salata Institute for Climate and Sustainability, Harvard University, Boston, MA, USA; 6 Department of Social Work, Faculty of Medicine, Dentistry and Health Sciences, University of Melbourne, Parkville, Victoria, Australia

## Abstract

Extreme heat poses a growing threat to vulnerable urban populations, and the existing heat early warning system usually operates at population level. Pairing emerging individualized and population early warning systems could directly and meaningfully extend protection to those most in need.

Climate change is amplifying the frequency, intensity and duration of extreme heat events. As global populations age and urbanize^1^, older adults, especially those with heat-sensitive chronic diseases (for example, cardiovascular disease), face heightened risks of heat-related morbidity and mortality^2^. Age-related impairments in thermoregulatory mechanisms, such as reduced skin blood flow and sweating, often coincide with declines in cardiovascular and kidney function, and increased basal inflammation, making extreme heat not just a thermoregulatory challenge but an all-systems stressor in ageing populations.

An individual’s ability to protect themselves from the adverse effects of hot weather depends on previous awareness of impending heat events and the capacity to implement appropriate protective actions. Effective behavioural responses require a recognized need to act, which is influenced by risk awareness and understanding of physiological status, along with the interplay between physiological and cognitive responses to heat stress^3^. Although older adults exhibit heightened vulnerability to heat, they often do not consider themselves at risk of the health dangers associated with extreme heat exposure^4^.

Most existing heat early warning systems (EWS) operate at the population level, using broad meteorological thresholds to issue alerts. While effective at scale, they lack sensitivity to individual variability in heat vulnerability and personalized alerts. Recent advances in wearable biosensors^5^, digital health technologies and advanced predictive modelling^6^ enable more targeted EWS^7–9^. These individualized heat EWS offer the potential to motivate protective behaviour by closing the perception gap for those who might otherwise underestimate their susceptibility to heat exposure. However, these innovations are not without limitations. The cost, digital literacy requirements and unequal access to reliable Internet or mobile infrastructure may limit uptake, especially among lower-income or technologically sceptical older adults. As such, while individual EWS offer considerable promise, ensuring equitable access and affordability must be a central focus in their design and deployment.

## Current population-based EWS

Population-level EWS are a central component of heat-health action plans for enhancing heat preparedness, reducing morbidity and mortality during periods of hot weather^10^. Population-based EWS use meteorological forecasts to issue timely alerts, encouraging protective behaviours across entire communities. Their effectiveness is well supported by evidence^11^. For example, in Milwaukee, USA, the implementation of an EWS was associated with a 49–73% reduction in emergency medical dispatches on heatwave days. In Philadelphia, USA, it was estimated that EWS saved 117 lives over 3 years, yielding a cost–benefit ratio of nearly US$468 million in saved lives compared with US$210,000 in operational costs. While some variability exists depending on the local context, climate, definition of a heatwave and population, these systems are recognized by the World Meteorological Organization and the United Nations as critical components of climate adaptation and disaster risk reduction^9^. Continued evaluation and refinement are necessary, but the foundational role of population-wide EWS in public-health response is clear.

**Fig. 1:**
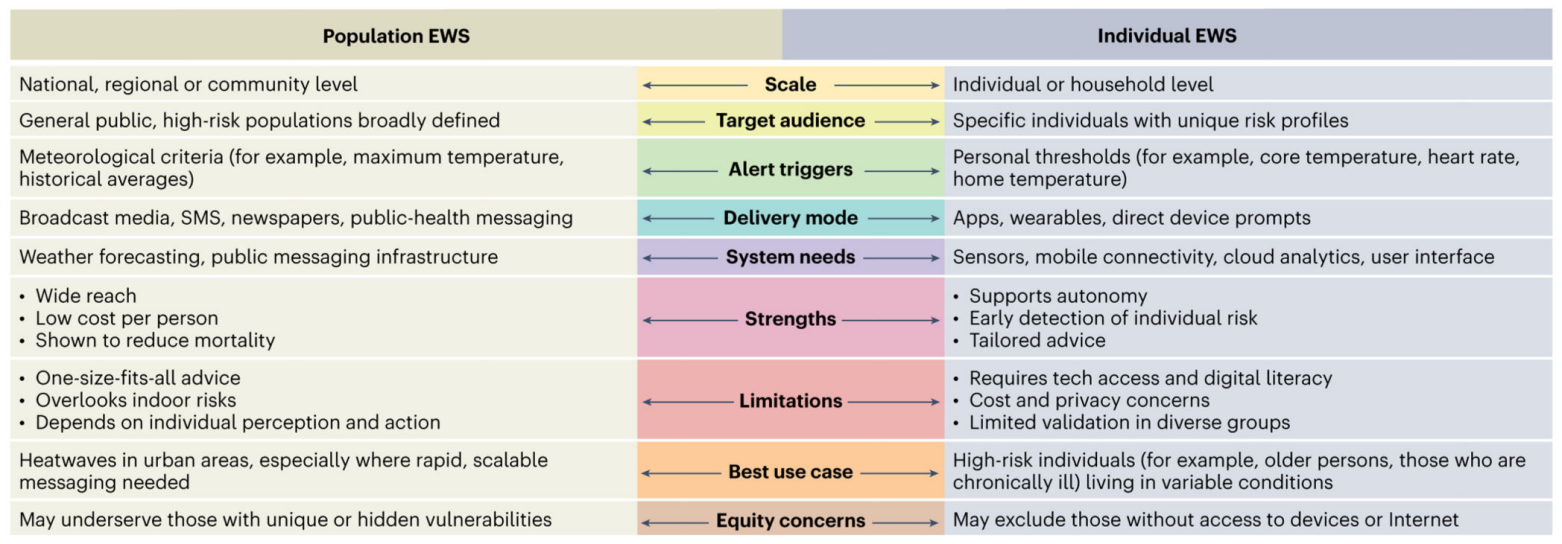
Distinctions and characteristics of population and individual heat EWS. Comparison of population-level and individual-level EWS for heat, highlighting differences across key domains.

However, population EWS are not without their limitations (Fig. 1). Given urban spatial heterogeneity (for example, green spaces and building density), population-wide EWS often inadequately capture the varied exposures experienced by urban populations, particularly those living in socioeconomically disadvantaged neighbourhoods. It is well established that cities experience higher temperatures than rural areas (termed the urban heat island effect), the intensity of which is unevenly distributed. For instance, analyses of the 2021 Pacific Northwest heatwave revealed that individuals from materially and socially disadvantaged backgrounds were approximately three times more likely to experience heat-related mortality^12^. Critically, many at-risk populations (that is, older adults and those with chronic diseases and/or disability) often come from these vulnerable backgrounds^13^ and consequently reside in areas disproportionately affected by elevated urban temperatures. For these individuals, experienced temperatures are often uncoupled from meteorological forecasts and triggers of population EWS. While population-wide EWS are invaluable for reducing heat-related harm at scale, they are inherently limited in their ability to capture individual variability in heat exposure and subsequent vulnerability.

## Emergence of individualized EWS

Existing population EWS could be further strengthened by incorporating individualized components, such as wearable technologies and personalized alerts and cooling recommendations, to better account for indoor exposures, physiological differences and varying adaptive capacities, ultimately enhancing the reach and responsiveness of population-wide efforts. Emerging evidence suggests that individualized EWS, which can account for personal physiological differences and microclimate exposures, add an essential additional layer in how to predict, communicate and respond to heat stress. Ever-improving wearables^5^, digital health technologies (for example, smartphone applications and cloud-based computing), advanced modelling^14^ and machine learning^6^ offer a promising suite of tools for enabling continuous, real-time monitoring and prediction of individual heat responses. Coupling physiological signals with localized environmental data can yield personalized risk assessments and precise intervention triggers, tailored uniquely to an individual’s health status and heat tolerance thresholds. Emerging research highlights that such individualized approaches can detect subtle deviations in predicted^6^ and measured^8^ physiological parameters, allowing proactive rather than reactive responses^4^.

Where individualized EWS surpass traditional approaches is through their enhanced capability to interact directly and meaningfully with the user, translating microscale environmental and physiological data into real-time behavioural nudges, prompting personalized cooling and heat mitigation strategies. Unlike standard public advisories, which may be overly broad, misaligned with user needs and not reach the most vulnerable, personalized alerts can offer actionable guidance that reflects the individual’s health status and available resources. For example, advice to engage in active cooling (for example, taking a cold shower or using a fan) can be tailored based on whether the individual is already exhibiting early signs of heat strain, has health and/or mobility limitations that contraindicate certain methods of heat mitigation, or lives in a setting where access to water or electricity is constrained. If indoor sensors show that a living space is the hottest part of the home, the system could prompt the user to move to a cooler space while notifying family members or caregivers of real-time environmental conditions and subsequent heat-health risk. This outsourcing of risk appraisal, shifting the cognitive and perceptual burden of heat risk assessment from the individual to an intelligent system, has the potential to empower users who may otherwise underestimate their vulnerability, such as those with cognitive or sensory limitations.

Individualized EWS are not simply a panacea for mitigating heat-health risk (Fig. 1). Equity remains a major concern. The technologies that underpin individualized EWS (that is, wearables, mobile phone apps and Internet connectivity) may be inaccessible to those in lower socioeconomic groups or with limited digital literacy. Data privacy and trust in algorithmic decision-making are also concerns, particularly when physiological monitoring becomes more invasive or continuous. Furthermore, while emerging evidence is promising^4,8^, thorough clinical validation of these systems is needed, and it is currently unclear how they perform in diverse populations or in individuals with underlying health conditions.

## Advancing EWS

To effectively mitigate the rapidly escalating risks associated with climate change-driven extreme heat, future research and policy must evolve along several key pathways. Firstly, high-resolution weather forecasts, satellite-derived urban heat maps, hospital admissions and demographic vulnerability indices all exist in the public domain, just rarely in the same place. A priority for research consortia is to build open, privacy-secure platforms that integrate these streams, with optional inputs from physiological and household monitoring to accurately predict and alert individual-level vulnerability. Crucially, the resulting alerts should travel back to communities or individuals via the channels they already use such as push notifications where smartphones are common, SMS, automated voice calls, or radio broadcasts where digital access is sparse. This necessitates transdisciplinary collaboration between climate scientists, physiologists, epidemiologists, communication specialists and technologists.

Secondly, it is imperative that the lived experiences of those most vulnerable to heat-health impacts are included in the design of individualized heat EWS. Embedding the beneficiaries (for example, older adults and those with disabilities or disease), their formal and informal carers, and frontline health workers in co-design yields communication pathways and solutions that are trusted, accessible, culturally appropriate and action-oriented.

Thirdly, international policy guidance, aligned with the World Meteorological Organization’s ‘Early Warnings for All’ framework^9^, will help to codify best practice while allowing for localized adaptation (for example, alert thresholds, colour codes for risk and advice).

Finally, research funding should prioritize scalable pilot studies and real-world trials evaluating individualized EWS and their inherent features, comparing outcomes across different technological approaches. Key research priorities should include identifying effective delivery modes (low tech versus high tech), measuring health and economic outcomes, and assessing metrics related to affordability, trust, equity and user acceptability. Alongside these studies, further work is needed to inform broader strategic actions of EWS as a whole, including:

- Defining minimum standards and best practices for scalable interventions (for example, cooling refuges^14^), considering governance models, accessibility and resilience during compound crises (for example, power outages, fires and floods).
- Developing harmonized risk thresholds and coordinated response protocols across local, state and federal government levels.
- Facilitating policy coherence and integrated action across health, housing, energy and emergency-management sectors.

## Conclusion

Extreme heat is no longer a sporadic seasonal threat but a chronic pressure undermining the health of the world’s fastest-growing, oldest and sickest (for example, those with multi-morbidities) urban populations. Pairing robust, population-based and individual EWS, physiological and environmental monitoring, and equitable, context-specific cooling options offers a promising path to climate-resilient communities. While fully coordinated investment across governments, meteorology, public health, thermophysiology, urban planning and digital technology remains a longer-term goal, immediate progress is achievable through identifying and targeting improvements in existing heat-health action plans, including population-based EWS. In the short term, identifying practical, actionable and achievable measures within current frameworks^9^ is essential to protect vulnerable populations effectively.
